# The Anatomy of Sleep, or the Art of Procuring Sound and Refreshing Slumber at Will

**Published:** 1843-01

**Authors:** 


### ART. XVI.

*The Anatomy of Sleep, or the Art of procuring sound and refreshing Slumber at will.* By EDWARD BINNS, M.D. &c.—*London,* 1842. 8vo, pp. 394.

THIS is a comical, pantosophical, popular sort of book, the author of which appears to be in extremely good humour with himself and everybody else. Its more immediate object is to communicate to the world the important secret of procuring sleep at will. Dr. Binns lays no claim to the discovery of this secret, which he attributes to the late Mr. Gardner, but has thought fit to accompany its announcement with a copious inquiry into the philosophy of sleep; and has produced a volume which, though its subject be sleep, and though it bear on its title-page a four-post bedstead with sumptuous hangings, has decidedly no hypnotic tendency during the perusal, but keeps us wide awake by a succession of wonderful narrations and posing questions, and a variety of matter embracing everything from metaphysics to cosmetics.

Dr. Binns commences by defining sleep as "the art of escaping reflection,"—a definition which we consider very faulty, first, because sleep is not an art, and secondly, because reflection is not always entirely banished during sleep. Precision in the use of terms is by no means one of our author's characteristic excellences: he here calls sleep an *art;* in another place he calls sleep a *property*, a *faculty*, and death a *quality*.

In the second and third chapters, and the thirteenth, which in natural sequence should have followed them, a popular view is taken of the organization, functions, and classification of living beings, which may prove in some degree instructive to the general reader.

In chapter the fourth the author makes some remarks on the physiology of *natural sleep*, and the subjects of dreaming and somnambulism being touched upon, lead to that of *animal magnetism*. The observations of the "Times" on the exhibitions of M. Lafontaine, and those of the "Medical Times" on the experiments of Dr. Elliotson, are quoted at full length. Dr. Binns informs us that he himself witnessed the performances of the first-named gentleman "in silent wonder not unmingled with incredulity,"—a very proper frame of mind on such an occasion.

Chapter the fifth treats of *prolonged sleep* and *trance*, and contains the alarming announcement that many persons are annually buried alive —which we slightly doubt. That such an event has occasionally happened is, however, but too true, and our author cites a number of instances, some of a horrible and others of an interesting or ludicrous character.

Chapter the sixth treats of *hybernation*, and the dormant vitality of animals and vegetables, but contains merely the common-places of these subjects. In the succeeding chapter Dr. Binns reverts to *somnambulism*. He adopts the opinions of Gall with respect to the physiology of that state, and cites various cases illustrative of its phenomena. The eighth chapter is on *catalepsy*, and consists almost entirely of cases,—some properly referrible to this head and others not so. Chapter the ninth is devoted to the subject of dreaming. It contains numerous anecdotes of extraordinary dreams and coincidences, and some sufficiently sensible remarks on the rationale of such occurrences, none of which, however, have any claim to originality. Chapter the tenth is on *hallucination*, and is of a very rambling character. It begins by defining hallucination as "a difficulty of feeling or appreciating external relations with which we are in connexion." We must profess our inability to comprehend the meaning of being in connexion with a relation, seeing that connexion is a relation. The examples adduced by Dr. Binns are extremely heterogeneous, involving various cases of insanity and depravation of the external senses, and one, at p. 231, in which we cannot trace any hallucination, but simply the natural effects of intense grief in a person of strong attachments. The next chapter is on *fainting*, *asphyxia*, and the *various modes of death*. The commencement of this chapter affords a remarkable exemplification of the inaccurate use of terms which is one of our author's besetting sins:

"As that temporary, or sometimes prolonged, or often fatal interruption to the phenomena of life, called Fainting, a condition of the body which may be induced by terror, exposure to mephitic gases, hemorrhage, suffocation, suspension or strangulation, and called by medical writers Syncope, or Asphyxia, literally signifying want of pulse, cannot be classed among bodily diseases, and as it is analogous to trance, and in fact, differs from that affection only in degree, it will require a short notice at our hands." (pp. 257-8.)

Among the numerous striking narrations of the fate of those persons whose earthly pilgrimage has come to an äerial termination in front of the Old Bailey, we do not recollect to have ever seen the term "fainting" applied to the effects of suspension. We would suggest to the gentlemen of the daily press the occasional substitution of "swung off in a syncope" for "launched into eternity," the latter phrase having become stale and vapid through frequent iteration. The popular reader will meet in this chapter with some information as to the several ways in which our "fiery particle" may be extinguished, and divers wonderful instances of resuscitation after apparent death.

On the subject of *mesmerism*, treated by Dr. Binns in his twelfth chapter and elsewhere, we shall not at present dwell, as we may possibly ere long enter into it in detail. We must, however, protest against such precipitate conclusions as the following, which we find in the sixteenth chapter:

"It cannot be too frequently repeated, that mesmerism depends upon the presence of a fluid passing from one body to another, and not to [upon] monotonism; and that the mesmeric state is consequently as distinct from that of normal sleep, as is the apoplectic stupor from refreshing slumber."

This position is supported by a very singular argument, amounting to as neat a *lucus à non lucendo* as could possibly be wished for:

"We infer the presence of this fluid from the fact, that 'if we examine with a microscope the naked body, exposed during summer to the rays of a burning sun, it appears surrounded with a cloud of steam, which becomes invisible at a little distance from the surface. And if the body is placed before a white wall, it is easy to distinguish the shadow of that emanation.' This is *not* the mesmeric fluid; but the time *may not* be far distant when the ingenuity of man may contrive a mesmerometer, as we have already electrometers, and that fluid be known also not only from its effects but from its composition; and the problem be solved whether it be a simple or a compound body." (pp. 375-6.)

The author adds in a note, "The word fluid is used not in its general sense, but we can find no other word to express conveniently mesmeric influence." But if we are to hope that we shall one day be able to ascertain the composition of this thing (so we must call it for want of a better term), that hope can rest only on the conviction that it is material; again it is pretty clear, admitting it to have any existence at all, which we neither affirm nor deny, that it is not a solid, and if so what can it be but a fluid?

In chapter the fourteenth the seat and constitution of the mind are considered. Dr. Binns adheres to the phrenological view of the cerebral functions, and concludes in a manner which engenders the hope that we are at length approaching the proper subject of this very comprehensive publication.

"From these cases, and the facts and arguments which have preceded them, it will be seen that the brain consists of a congeries of organs, whose functions are especially distinct and appropriated for particular purposes; that it is the seat of the intellectual powers; that in it essentially resides the principle of vigilance or wakefulness; and that we must therefore look for the organ or faculty of sleep in some other part of the body; and with a view to facilitate inquiry, we shall proceed, in the next chapter, to the description of SLEEPLESSNESS OR VIGILANCE."

In the two concluding chapters of the work the actual nature of sleep and the means of inducing it are considered. Dr. Binns regards sleep not as the suspension of certain functions, but as a positive state resulting from the activity of a certain portion of the nervous system. We give his views in his own words:

"That the state of fatigue in any particular organ is not exactly the same as its condition in sleep, we infer from the fact, that over-fatigue or over-excitement, or excitement ever so trifling of the voluntary organs, if we happen not to be accustomed to it, will often prevent instead of predisposing or inducing sleep. Again, we sleep periodically and at stated times, whether fatigued or not, while many possess the faculty of sleep at will, at all times and all places. We are told by Bourrienne that when Napoleon was at Tilsit, sitting in a chair next the Emperor Alexander of Russia, and witnessing the performance of the Cid of Corneille, suddenly in the very midst of the representation, as if wishing to abstract himself from the mimic scene before him, folded his arms, reclined his head upon his chest, and was sound asleep. Here was the exertion of a faculty—a power distinct from the mere condition of fatigue; it was functional manifestation of some organ within the body. But where is this organ situated? In the cerebrum, cerebellum, or medulla oblongata? We think in no one of these organs, but that we are to look for it in the medulla spinalis or spinal cord, and that it will be found between the cervical and lumbar vertebræ in the GANGLIA formed from the nerves given off by this portion of the spinal column. The reasons which induce us to place it in this region are, that sleep is intimately connected with respiration, that the true functions of the spinal cord have never been satisfactorily ascertained; that the ganglionic system is the antagonism of the cerebral; and lastly, that no phenomena can arise, or can manifest themselves in the body purely from the cessation of activity of any organs, but must owe their presence to some direct cause." (pp. 385-6.)

It is the true saying of Samuel Johnson that "on the arena of conjecture all men stand equal who are equally well informed;" and on this principle we leave Dr. Binns in quiet possession of his theory.

It has long been known that monotony, or a continued repetition of similar impressions on the mind or senses, was a powerful agent in producing sleep; but according to our author, this was first reduced to a principle by Mr. Gardner. The results obtained by Duncan, Braid, Catlow, and others, are all referred by Dr. Binns to this principle, which he calls *monotonism:* he considers such phenomena as belonging to those of "enhanced normal sleep," and by no means as identical with those of mesmerism, which arise from the agency of a pecular fluid. But it would be tantalizing the reader to withhold from him any longer the *arcanum magnum.* Here it is! Suppose a man cannot sleep:

"Let him turn on his right side, place his head comfortably on the pillow, so that it exactly occupies the angle a line drawn from the head to the shoulder would form, and then slightly closing his lips, take rather a full inspiration, breathing as much as he possibly can through the nostrils. This, however, is not absolutely necessary, as some persons breathe always through their mouths during sleep, and rest as sound as those who do not. Having taken a full inspiration, the lungs are then to be left to their own action; that is, the respiration is neither to be accelerated nor retarded. The attention must now be fixed upon the action in which the patient is engaged. He must depict to himself that he sees the breath passing from his nostrils in a continuous stream, and the very instant that he brings his mind to conceive this apart from all other ideas, consciousness and memory depart; imagination slumbers; fancy becomes dormant; thought subdued; the sentient faculties lose their susceptibility; the vital or ganglionic system assumes the sovereignty; and as we before remarked, he no longer wakes but sleeps." (p. 391.)

This plan, Dr. Binns tells us, has never failed, so far as he knows, but in two instances. We are happy to say that our own proclivity to repose at the proper hour, (enhanced, no doubt, by the peacefulness of our editorial conscience, which allows us "to sleep in spite of thunder" from Dr. Paine and other malcontents,) has not given us an opportunity of trying Dr. Binns's soporific; some of our friends, however, have made a few trials of it, but have not found it succeed in one. Such are the diversities of human experience! We think it very probable, however, that the respiration may be so regulated as to favour sleep, and we recommend the subject to the attention of our readers.

Such are the general contents of Dr. Binns's book, and we wish we could dismiss it without noticing the inaccuracies both as to facts and references with which it abounds. The author seems generally to cite from memory, a very dangerous practice, unless the memory be an unusually good one, as will appear from the following examples, to which many others might be added. At p. 121, Byron's "sentimental savage," as applied to Isaac Walton, is changed into "sentimental sinner," and the humour of the expressiou thereby destroyed. At p. 279 the poet Cowley is made to hang himself over his chamber-door in the Temple. Dr. Binns here confounds Cowley with Cowper; the former never lived in the Temple, nor, as far as the world knows, ever attempted suicide. At p. 371, the experiments and views of Dr. Kellie with respect to the cerebral circulation are attributed to Dr. Abercrombie. &c. &c.

With all its defects, however, we think it but just to say that the "Anatomy of Sleep" is an entertaining book, involving a variety of general information. We have no doubt that it will be read by many with much amusement, and not without some edification.

